# Molecular Combing of Single DNA Molecules on the 10 Megabase Scale

**DOI:** 10.1038/srep19636

**Published:** 2016-01-19

**Authors:** Atanas Kaykov, Thibaud Taillefumier, Aaron Bensimon, Paul Nurse

**Affiliations:** 1 The Rockefeller University, 1230 York Avenue, New York, NY 10065, USA; 2 Lewis-Sigler Institute for Integrative Genomics, Princeton University, Princeton, New Jersey, USA; 3 Genomic Vision, 80-84 rue des Meuniers, 92220 Bagneux, France; 4 The Francis Crick Institute, Lincoln’s Inn Fields Laboratories, London WC2A 3LY, United Kingdom

## Abstract

DNA combing allows the investigation of DNA replication on genomic single DNA molecules, but the lengths that can be analysed have been restricted to molecules of 200–500 kb. We have improved the DNA combing procedure so that DNA molecules can be analysed up to the length of entire chromosomes in fission yeast and up to 12 Mb fragments in human cells. Combing multi-Mb-scale DNA molecules revealed previously undetected origin clusters in fission yeast and shows that in human cells replication origins fire stochastically forming clusters of fired origins with an average size of 370 kb. We estimate that a single human cell forms around 3200 clusters at mid S-phase and fires approximately 100,000 origins to complete genome duplication. The procedure presented here will be adaptable to other organisms and experimental conditions.

Single molecule studies have grown in importance because they allow investigation of individual molecules whose characteristics may vary greatly from molecule to molecule and from cell to cell. Traditional ensemble approaches analyse the entire population of molecules averaging their characteristics, which does not take this variability into account. DNA combing stretches single DNA molecules uniformly at high density on silane coated glass surfaces^1^,^2^. This method is used routinely for studying DNA replication^3^,^4^,^5^, DNA–protein interactions^6^, *in vitro* transcription^7^, genomic rearrangements^8^,^9^, and analysis of repetitive sequences^10^,^11^, which are difficult to investigate with DNA sequencing techniques. DNA combing does not require modification of DNA, and uses the surface tension of a receding water-air interface to extend and immobilise single DNA molecules on a solid surface. Furthermore, DNA molecules are labelled in their cellular context, an advantage over *in vitro* replisome reconstitution methodologies^12^, that have been used to analyse DNA replication on single DNA molecules but outside the cell.

DNA replication initiates at origins of replication located at specific sites along chromosomes and cells utilise a subset of available origins to complete genome duplication^13^. Population based approaches average the frequency of origin usage and replication fork velocities^14^,^15^. DNA combing has been used to investigate the pattern of replication origin firing and replication fork velocities on genomic single DNA molecules^4^,^10^,^16^,^17^,^18^. This reveals cell-to-cell differences in origin usage important for understanding how genomes are replicated during S-phase. However such studies have been limited because only relatively short DNA molecules of 200–500 kb can be analysed by DNA combing technique, which restrict the analyses to small segments of chromosomes.

Here we report a modified DNA combing protocol that allows the analysis of DNA replication on single DNA molecules up to 12 Mb in length. We have developed this methodology in the fission yeast *Schizosaccharomyces pombe* where we can analyse molecules whose length average is 2 Mb with some molecules occasionally reaching 5.6 Mb, allowing the entire length of all three fission yeast chromosomes to be investigated. We have extended our new combing method to human U2OS cells and have analysed single DNA molecules on average 8 Mb in length and occasionally up to 12 Mb. Changing the scale of analysis of replicating single DNA molecules by more than an order of magnitude reveals a more extended structure of origin firing. Replication origins fire stochastically and form clusters of closely spaced origins separated by regions of sparsely firing origins. Our findings suggest that the spatial organisation of fired origins is similar in different eukaryotes.

## Results

### DNA combing methodology

**Figure 1:**
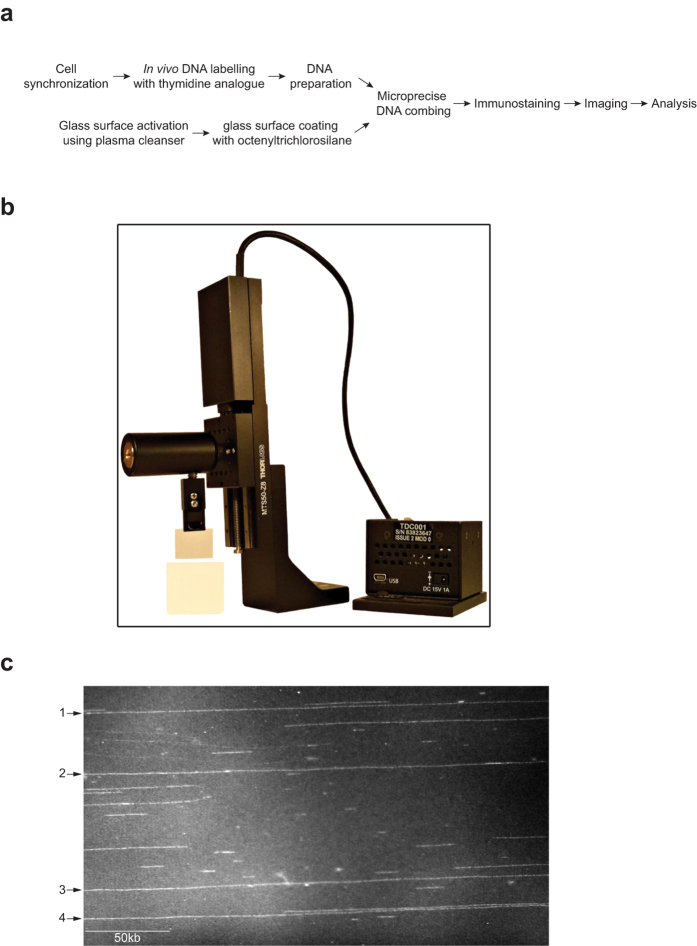
DNA combing. (**a**) DNA combing workflow chart. (**b**) DNA combing machine assembled using high precision mechanical modules and adjustable speed. This allows precise movement along the vertical axis with minimal vibration, which helps reduce shearing of DNA molecules. (**c**) Representative image of YOYO-1 stained DNA molecules acquired with epifluorescent microscopy. Four single DNA molecules span the field of view (indicated with arrows).

We used DNA prepared from fission yeast to develop improvements in the DNA combing procedure that can extend the length of molecules that can be analysed. DNA combing requires stretching, aligning and immobilizing protein free DNA molecules on hydrophobic surfaces such as silane or PDMS^1^,^19^. To prepare the surface, glass slides were activated in a plasma cleanser to make the glass chemically reactive. The activated slides were then placed in a gas chamber saturated with octenytrichlorosilane vapour, which reacts with the activated glass resulting in a silane-coated glass surface (Fig. 1a). Fission yeast genomic DNA was labelled *in vivo* with thymidine analogues such as BrdU or EdU in a synchronous cell population (Supplementary Fig. S1a), and prepared in agarose blocks which were melted and digested with β-Agarase releasing protein-free DNA molecules into solution (Fig. 1a). DNA molecules in solution bind to the vinyl group (–CH = CH2) of the silane at a pH range between 5.5 to 6.5^20^. Pulling the slide from the solution exerts a force antiparallel to the movement of the glass slide that uncoils and stretches the DNA molecule along the slide (Fig. 1b). The DNA molecules are immobilized by dehydrating the surface, and can be stained with intercalating dyes such as YOYO-1 for imaging by epifluorescent microscopy (Fig. 1c).

Previous combing protocols break chromosomes into pieces of 200–500 kb in length^2^,^4^,^21^. To reduce DNA breakage we reasoned that since full-length fission yeast chromosomes up to 5.6Mb in length can be resolved in pulse field gel electrophoresis (DNA is also prepared in agarose blocks for PFGE^22^ as for DNA combing), DNA molecules prepared for combing are likely to be breaking after melting the block and/or during the combing process itself, but not during DNA preparation. The force applied by the receding meniscus of the air-water interface to the single DNA molecule was estimated to be around 160pN^23^, a force approximately 10 times lower than that is needed to break a covalent bond (~2 nN depending on the nature of the covalent bond)^24^. This suggested that DNA breaks are likely to occur at an earlier step such as during the pouring of the melted agarose blocks into the combing reservoir. Therefore we removed this processing step by directly melting agarose blocks into the combing reservoirs and then adding β-Agarase. This very much improved the length of molecules that could be combed and imaged to the several Mb range. However, such long single DNA molecules were not abundant and the majority of DNA fragments were still only several hundred kb in length.

To improve the effectiveness of DNA combing method we considered the factors that could influence the mechanical stability of double-stranded DNA (dsDNA) molecules such as the ionic strength and pH of the combing buffer, the temperature, the base composition and the pulling velocities exerted on the DNA molecule^25^,^26^,^27^,^28^. The force applied to single DNA molecules by the water-air interface overstretches DNA molecules to 1.6 times its contour length^23^,^29^. Applying a force that overstretches a single dsDNA molecule to 1.6 times its contour length induces melting of the dsDNA molecule generating single-stranded DNA (ssDNA) molecules^25^,^26^. This suggests that the melting of dsDNA might occur during combing. We reasoned that if closely spaced single stranded nicks are present on two complementary strands separated by a short distance, the melting of this portion of the molecule could result in the loss of the DNA molecule which is still in the solution and thus result in “broken” shorter DNA molecules after combing^29^. We introduced modifications to these factors, aimed at preserving the length of replicating single DNA molecules that can be combed by reducing DNA shearing. First, we constructed a DNA combing machine which generated smooth, low friction movement with limited vibration (Fig. 1b and Supplementary Fig. S6). Second, as already described, we directly melted the agarose blocks in the combing reservoir, which decreased shearing. Third, DNA molecules were combed in a buffer containing 100 mM of NaCl with a pH of 6.0^27^. At this NaCl concentration, Na^+^ ions effectively screen the negatively charged phosphate groups along the backbone of the dsDNA molecules to reduce electrostatic repulsion and melting of dsDNA, thus reducing the fraction of broken DNA molecules^26^,^29^. The density of arrayed DNA molecules is also enhanced at 100 mM NaCl, when compared to DNA combing at pH 6.0 without NaCl^19^. Fourth, we lowered the temperature after β-Agarase incubation to room temperature before DNA combing to reduce melting of dsDNA, as it has been shown that at 40 °C dsDNA melts to ssDNA at lower stretching forces^26^. Fifth, we adjusted the combing speed to 900μm/second as it has been shown that the optimal motion rate of the water-air interface is around 900μm/second resulting in uniformly stretched DNA molecules with minimum breakage^28^. Sixth, we prepared genomic DNA in agarose blocks using a minimal incubation time (see Supplementary Methods) ensuring that almost all cells were intact in the solidified agarose block prior to cell breakage. To estimate the cumulative effect of all these changes we plotted the fraction of DNA molecules of different lengths observed under the microscope (Supplementary Fig. S2a). It can be seen that 48% of the molecules are longer than 1 Mb. Using this modified protocol we could comb DNA molecules from fission yeast on average of 2 Mb in length and occasionally up to whole chromosomes of around 5.6Mbf in length^30^.

### Clusters of fired origins can be characterised on single multi-Mb-long DNA molecules

**Figure 2:**
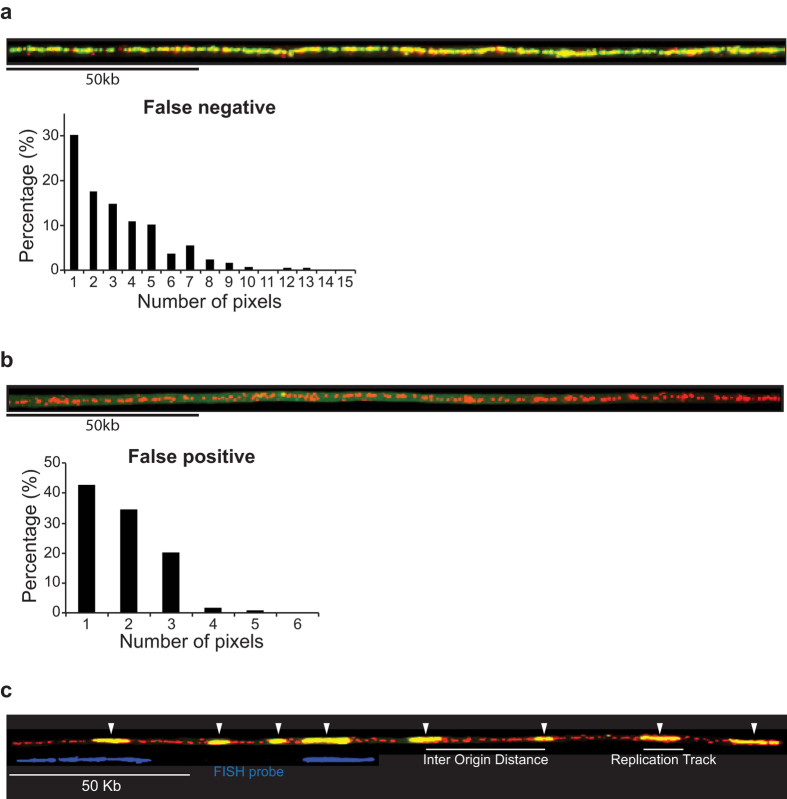
Combing full-length fission yeast chromosomes. Fission yeast genomic DNA was labelled *in vivo* with BrdU using a synchronous cell population. Genomic DNA was prepared for DNA combing from cells at mid S-phase. Single DNA molecules immobilized on glass slide were denatured and BrdU incorporated into newly synthesised DNA was visualised with fluorescently labelled anti-BrdU antibody (green). The whole DNA molecule was counterstained with anti-single stranded DNA antibody (red). (**a**) A representative DNA molecule completely replicated in the presence of BrdU is shown. The quantification of false negative staining, corresponding to the gaps in the BrdU signal expressed as number of pixels is shown below the molecules. Ten DNA molecules corresponding to 5Mb of DNA were analysed. We used a gap size threshold of ≤10 pixels to identify false negative staining in the segmentation of BrdU tracks. (**b**) A representative unlabelled DNA molecule stained with anti-single stranded DNA antibody (red) and anti-BrdU antibody (green) is shown. The quantification of false positive staining, corresponding to green dots on the molecules expressed as number of pixels is shown below the molecule. Ten DNA molecules corresponding to 7 Mb of DNA were analysed. We used a threshold of >3 pixels to identify replication tracks. (**c**) An example of DNA molecules undergoing DNA replication is shown. White arrows point to the middle of replication tracks (shown with a short white line below the DNA molecule) where replication origins are most likely located. The inter origin distance represents the distance between the middle of two adjacent tracks. Biotinylated FISH (fluorescent *in situ* hybridisation) probes (shown in blue below the molecule) were used to align DNA molecules to their corresponding chromosomal sequences. DNA probes were designed with unique signature left and right arms and a gap between them, allowing the simultaneous detection of different chromosomal loci.

We used the new DNA combing method to investigate the pattern of replication origin firing on multi-Mb long single DNA molecules in fission yeast. We first synchronised cells for entry into S-phase and pulse labelled newly synthesised DNA with BrdU (Supplementary Fig. S1a,b). Genomic DNA was then combed onto silanized glass surfaces and immunodetected. Newly synthesised DNA incorporating BrdU is shown in green, with whole molecules counterstained with anti-thymidine antibody are shown in red (Fig. 2a–c). To accurately segment BrdU tracks along the DNA molecules two control experiments were performed to determine the number of pixels corresponding to false negative and false positive staining. False negative staining was quantified using combed DNA molecules from a cell population that was labelled with BrdU for an entire cell cycle (Fig. 2a). We found that BrdU staining can exhibit gaps and that 98% of these gaps are no longer than 10 contiguous pixels (but are partially labelled with the anti-single stranded DNA antibody (histogram Fig. 2a)). Therefore to exclude false negatives, we did not take into account gaps in BrdU staining of less than 10 pixels. To quantify false positive staining we combed genomic DNA prepared from an unlabelled cell population and stained with an anti-single stranded DNA antibody and an anti-BrdU antibody (Fig. 2b). We found that occasionally BrdU staining could be detected, however 98% of these false positive staining were not more than 3 contiguous pixels (histogram Fig. 2b). Therefore to exclude false positives, replication tracks were only measured for 4 or more contiguous pixels. These empirically established limits were used to segment replication tracks. Replication origins were mapped in the middle of individual replication tracks corresponding to replicons, and inter-origin distances (IODs) were estimated by the distances between midpoints of two adjacent replication tracks (Fig. 2c). We used FISH to identify the chromosomal regions being analysed (blue staining on Fig. 2c).

Comparing replication patterns on long DNA molecules imaged across consecutive microscopic fields of view requires *in silico* manipulation. We therefore developed an algorithm that enables representation of long, partially replicated DNA molecules as “barcode” diagrams, where stretches of replicated and un-replicated DNA are shown as coloured segments. These diagrams were built from the measurements of successive replicated and un-replicated DNA segments, together with a reference position on the chromosome determined by FISH. Due to the considerable number of measured segments, we used a machine-independent C routine that takes Excel data files as input to generate the bar code diagrams in Postscript vectorial format (see Supplementary Methods). Such barcode diagrams provide a convenient way to observe many different molecules at the multi-Mb scale aligned to corresponding chromosomal sequences identified using FISH.

**Figure 3:**
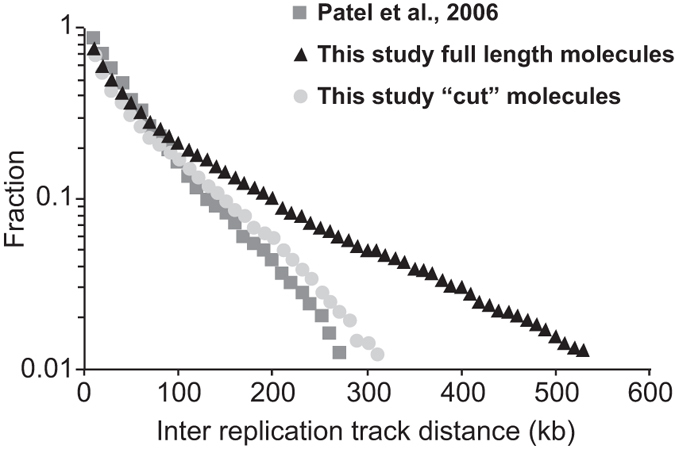
Mb-scale single DNA molecules reveal clusters of closely spaced fired origins. We plotted the cumulative frequencies of inter replication track distances (IRTDs) measured by Patel *et al*., 2006 (dark grey squares), the IRTDs measured on full length molecules analysed in our study (black triangles), and the same molecules analysed in our study but “cut” *in silico* to shorter 500 Kb fragments (light grey circles). The “cut” fragments show a similar distribution of IRTDs as the molecules analysed by Patel *et al*., 2006, indicating that the failure to detect clusters of closely spaced origins previously was due to the relatively short size of single DNA molecules analysed.

One 4 Mb partially replicated single DNA molecule is shown in Supplementary Fig. 2c. This DNA molecule was imaged across 15 consecutive microscopic fields, each encompassing 270 kb of the molecule, and a composite picture was constructed. The synthetic picture of the molecule is shown represented as “barcode” diagrams where green segments represent individual replication tracks and black segments represent un-replicated DNA (Supplementary Fig. S2c below the composite image). Replication origins fire in clusters of closely spaced origins separated by regions poor in fired origins^30^, which differs from an earlier study which reported that fission yeast fired origins were randomly located along chromosomes with no clusters^4^. We investigated whether this was due to the shorter molecules of 200–500 kb that had been analysed previously, by ‘cutting’ *in silico* our longer molecules into fragments of 500 kb and comparing their inter replication tracks distances (IRTD) with those analysed in the earlier study. Figure 3 shows that the distribution of IRTD cumulative frequencies was essentially identical between our cut molecules (light grey circles) and those analysed in the earlier study (dark grey squares), and can be fitted with a single straight line indicative of random origin selection with no clusters. This is different from the distribution of IRTD measured on long DNA molecules which can be decomposed into two straight lines with different slopes, indicative for clustering^30^. This analysis demonstrates that the clusters of fired origins can only be revealed on multi-Mb-scale single DNA molecules. Comparisons between different molecules aligned to the same chromosomal sequence showed that in different cells clusters were located in different positions^30^ and so could not be detected in population-based approaches such as micro-arrays or DNA sequencing^15^,^31^.

### Replication origin firing along human chromosomes

**Figure 4:**
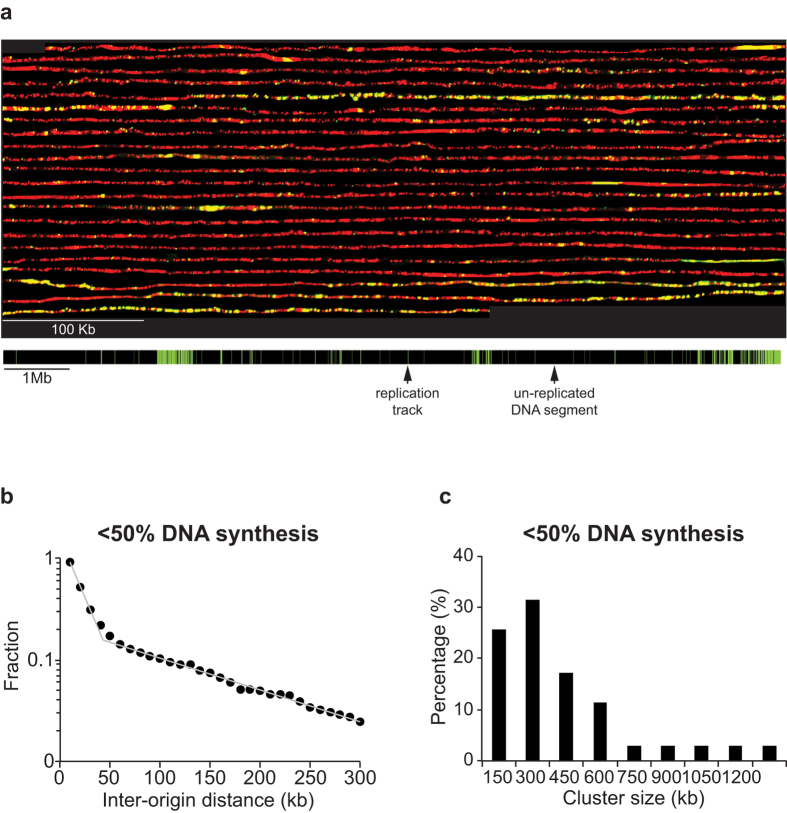
Replication origins fire stochastically and form clusters in human cells. (**a**) U2OS cells were synchronised and genomic DNA labelled *in vivo* by adding BrdU to the media before the onset of S-phase. DNA was prepared for combing from cells at mid-S-phase. BrdU incorporated into newly synthesised DNA was detected with fluorescently labelled anti-BrdU antibody (green) and the entire DNA molecule was counterstained with anti-single stranded DNA antibody (red). To visualise individual replication tracks, the molecule was “cut” *in silico* into 44 consecutive fragments of 270 kb each, and a composite picture was constructed. A representation of the entire 12 Mb single DNA molecule is shown below, with green and black bars corresponding to replication tracks and to un-replicated segments of the DNA molecule. Black and green bars are drawn to scale. (**b**) Semi-log plot of the cumulative frequencies of IODs for molecules replicated to 50%. The values on the y-axis correspond to the fraction of IODs that are larger in size than the corresponding IOD on the x-axis. The distribution of the experimentally measured values deviates significantly from a purely stochastic distribution, which is expected to fit a straight line (p < 0.001 Lilliefors statistical test). The Lilliefors statistical test is a non-parametric test for exponential distribution, which allows rejection of the hypothesis that the rate of origin firing is homogeneous along the sequence during the early stage of replication, without using any prior estimates from the data, such as the average firing rate. Instead, the cumulative frequencies of IODs lay on a plot with a complex shape that can be decomposed into two straight lines that cross at approximately 40 kb. The straight line with the steeper slope corresponds to IODs for origins firing stochastically at a higher rate within the clusters, whereas the straight line with the shallower slope corresponds to IODs firing stochastically at a lower rate within the regions between clusters (n = 577) (**c**) Histogram of cluster size for molecules replicated from 10% to 50% (45 Mb analysed).

We next extended our improved DNA combing protocol to organisms with larger chromosomes to determine the maximum length of DNA molecules that could be analysed. We investigated the distribution of origin firing in human U2OS cells, synchronising the cells by first blocking cell cycle progression at S-phase onset using thymidine followed by release and a second block in mitosis using nocodazole (Supplementary Fig. S3a). At the end of mitotic block we plated cells in fresh media, allowing them to enter the cell cycle with high synchrony and equilibrated nucleotide pools (Supplementary Fig. S3b). We added BrdU in G1 (5 hours after release), allowing genomic DNA labelling upon entry into S-phase. Cells were collected at mid S-phase (12 hours after release) and genomic DNA was prepared for DNA combing (Supplementary Fig. S3c). We imaged an average of 8 Mb long single DNA molecules using our improved protocol, and were able to identify DNA molecules up to 12 Mb, which is 25 times longer than previously reported for human cells^11^,^32^. An example of a long single DNA molecule is shown in Fig. 4a (“cut” *in silico* into 44 consecutive fragments) and the synthetic image of the whole DNA molecule is shown below. Visual examination showed that replication origins fire in clusters of closely spaced origins separated by long regions with sparsely fired origins. Clusters of closely spaced origins have been reported previously for Xenopus, mouse and human cell lines^33^,^34^,^35^.

To analyse the distribution of origin firing along human chromosomes we analysed 7 additional single DNA molecules averaging 8 Mb in length and replicated up to 50% (Supplementary Fig. S4a and S5b). The average replication track length on these molecules was 15kb (90% of replication tracks were shorter than 30 Kb), suggesting that the majority of replication tracks most likely stemmed from a single origin (Supplementary Fig. S4b). We investigated whether the observed uneven distribution of fired origins in human U2OS cells results from random origin firing by plotting the cumulative frequencies of IODs on a semi-log plot. If origin distribution were random, a straight line would describe the cumulative frequencies of IODs. Figure 4b shows that the data points form a plot with two clear domains, both of which can be fitted with straight lines with different slopes, indicative of stochastic origin selection operating in the two domains but with different rates. The segment of the plot with the steeper slope encompasses 80% of all fired origins, with an average IOD of 20 kb, and corresponds to origins firing within clusters. The segment of the plot with the shallower slope encompasses 20% of all origins fired with an average IOD of 125 kb, and corresponds to origins firing in regions between clusters. We conclude that replication origin selection in human cells operates at different stochastic rates along chromosomes, forming clusters of closely spaced origins separated by regions with sparsely fired origins.

Next, we characterised cluster size by setting the maximal IODs within clusters to be 40 kb, twice the average 20 kb IOD, and estimated the minimal number of origins per cluster by varying the number of origins defining a cluster from two to 15, and plotting it against the number of clusters per genome. Supplementary Fig. S4c shows that the descending segment of the curve reaches saturation at 6 origins, which we have assumed to be the minimum number of origins defining a cluster. We used the values of 40 kb maximal IOD and 6 origins minimum per cluster to delineate clusters along the DNA molecules. Figure 4c shows that 85% of clusters range in size from 150 kb to 600kb with an average of 370 kb. We estimated the number of clusters within a single U2OS cell to be around 3200 at mid S-phase and that each cluster contains on average 19 fired origins. Each cell has fired approximately 70 000 origins by mid S-phase. We conclude that although the average IOD within clusters is similar for human and fission yeast cells, cluster size in human cells is roughly twice the size of those in fission yeast cells^30^.

We also measured replication fork velocity in U2OS cells by chasing BrdU with EdU for 20 minutes at mid S-phase (Supplementary Fig. S5a). To derive replication fork velocities we measured the length of EdU tracks immediately adjacent to BrdU tracks and divided their length by the time of the chase (Supplementary Fig. S5b). Supplementary Fig. S5c shows that in U2OS cells fork velocities range from 0.5 kb/min to 2 kb/min with an average of 1 kb/min. These values are similar to previously reported fork velocities measured on single DNA molecules for this cell line^36^,^37^. We also estimated the total number of origins firing in a single U2OS cell to complete genome duplication by counting the number of fired origins on single molecules replicated to 85%, and normalising this number to the entire genome (example molecule 3 Supplementary Fig. S5b). We found that a single cell is using approximately 100,000 origins to replicate its genome, although this may be an underestimate because in DNA molecules that are almost completely replicated a number of tracks will result from merged replicons. This number is similar to reported estimates of 80,000 origins used per cells during each S-phase^38^. We conclude that replication origins fire stochastically along human chromosomes forming clusters of closely spaced origins separated by regions with sparsely firing origins.

## Discussion

We have modified DNA combing protocols to allow imaging of single DNA molecules up to 12Mb in length compared with previous limits of around 1 Mb. Our protocol allows analysis of origin firing and fork velocities on single DNA molecules genome-wide for organisms with small genomes and across large sub-chromosomal fragments in human cells. We showed that in human cells origin selection is a stochastic process forming clusters of closely spaced fired origins.

Previously, analysis of DNA replication on short DNA molecules resulted in different values for experimentally measured IODs and replication fork velocities for the same cell line^39^. These discrepancies may be due, in part, to differences in the sample size, the counterstaining of the entire DNA molecule, and the length of DNA molecules analysed in different studies. For example, the median IOD and the median fork velocities for DNA molecules with a median length of 104 kb, were 5-fold and 2-fold smaller than those measured for molecules with a median length of 378 kb^39^. Our improved DNA combing protocol could help to resolve these discrepancies since a relatively large sample of counterstained molecules can be imaged at the multi-Mb-scale.

Each cell must complete genome duplication in a timely fashion before the onset of mitosis. Failure to replicate even a small region of chromosomes would lead to genomic instability. Understanding the mechanisms underlying the timely completion of genome duplication will benefit from the analysis of models using experimentally measured parameters of the process of DNA replication. In organisms with small genomes and few chromosomes such as fission yeast, these parameters can be measured using DNA combing because a single DNA molecule at the scale of a full-length chromosome represents a significant part of the genome and can be used as a surrogate for the extent of DNA synthesis within the cell^30^. This allows an approach to estimate replication fork velocity and the rate of origin firing as an individual cell proceeds throughout S-phase.

DNA combing of multi-Mb-scale single DNA molecules allows the characterisation of the distribution of fired origins in clusters along human chromosomes. The absolute position of these clusters can be determined using FISH, allowing origins and clusters to be mapped to specific chromosomal sequences. In addition mutants and small molecules that perturb cluster characteristics can be screened to gain insights into their formation. Understanding the mechanisms responsible for cluster formation will bring insight to the higher order organisation of interphase chromosome in human cells. Finally, epigenetic modification such as DNA methylation can be detected on combed single DNA molecules using specific antibodies^40^. Using the protocol described here it should be possible to investigate the variability and inheritance of X chromosome inactivation, imprinted loci within the genome, transposon silencing and the repression of gene promoters on single DNA molecules. Ultimately, using specific reagents this method can be extended to analyse many kinds of DNA modification, such as ribonucleotide incorporation into DNA, single stranded nicks, or DSBs.

## Methods

### Fission Yeast Synchronisation and DNA Labelling

Standard fission yeast media and methods were used^41^. To obtain synchronised cell cultures, temperature sensitive *cdc25-22* strains were grown in minimal media (EMM) at 25 °C to 1–2 × 10^6 ^cells/ml, shifted to 36 °C for 4 hours to block cells in late G2, and then released at 25 °C. 2 μM BrdU was added to a synchronised culture at 34 minutes after release (corresponding to G1-phase) allowing cells to enter S-phase in the presence of BrdU. Cell cycle progression was stopped at 75 inutes after release (corresponding to 50% of DNA replication for the population, estimated from FACS profiles) and DNA was prepared for DNA combing (Supplementary Fig. S1a,b). The combing data analysed in this study are presented in Supplementary Table S2 and are derived from previously reported fission yeast measurements^30^.

### Human U2OS Cell Synchronisation and DNA Labelling

U2OS cells were cultured in DMEM containing 10% FBS at 37 °C with 5% CO_2_. To synchronise the population of cells in S-phase, cells were incubated in media containing 2.5 mM thymidine for 24 hours. Cells were then released in fresh media containing 100ng/mL nocodazole for 12 hours to block cell cycle progression in M-phase. At the end of mitotic block, rounded mitotic cells were shaken off and collected in fresh media. Five hours after mitotic release (corresponding to G1-phase for the population of cells, estimated from FACS profiles shown in Supplementary Fig. S3b) 10 μM BrdU was added to the cell culture. Cells were then collected at 12 hours after release (corresponding to mid S-phase for the population of cells, estimated from FACS profiles shown in Supplementary Fig. S3c) and samples were prepared for DNA combing. Alternatively, the BrdU label was chased with 30μM EdU for 20 minutes before samples were collected and prepared for DNA combing. The combing data analysed in this study are presented in Supplementary Table S1.

### DNA Combing

Genomic DNA was prepared for combing in 1% low melting agarose Mb grade plugs (Bio-Rad)^42^. The plugs were washed for two days in TE 1X pH7.5 with 100 mM NaCl (buffer changes), melted for 15 minutes at 70 °C in MES 50 mM pH = 6, 100mM NaCl, and incubated overnight with 2 μl β Agarase (New England Biolabs) without mixing at 42 °C in a Teflon reservoir (produced in the machine workshop at the Rockefeller University). Glass surfaces were activated in plasma cleanser (Harrick Plasma) and coated with 7-Octenyltrichlorosilane in a gas chamber. Genomic DNA was combed on silanized glass surfaces using a combing machine (assembled with products from Thorlabs) at a speed of 900 μm/sec. After DNA denaturation biotinylated DNA probes were hybridized to combed DNA. To position and orient the combed DNA molecules on different chromosomes, sets of two probes for each chromosomal region were designed with unique signature of differing lengths and distances between them. For each experiment two controls were processed; to quantify the number of pixel corresponding to false negative staining we used fully BrdU-labelled DNA and to quantify the number of pixel corresponding to false positive staining we used un-labelled DNA. Detailed description of DNA combing methodology is provided in the Supplementary Methods.

### Imaging and Quantification

Images for DNA molecules were collected in Metamorph (MDS Analytical Technologies) using an epifluorescence microscope (Axioplan 2, Carl Zeiss, Inc) equipped with a Zeiss Plan-FLUAR 63x/1.40 lense (Carl Zeiss, Inc) and CoolSNAP HQ camera (Roper Scientific).

## Additional Information

**How to cite this article**: Kaykov, A. *et al*. Molecular combing of single DNA molecules on the 10 megabase scale. *Sci. Rep.* **6**, 19636; doi: 10.1038/srep19636 (2016).

## Supplementary Material

Supplementary Information

Supplementary Dataset 3

Supplementary Dataset 4

Supplementary Dataset 2

Supplementary Dataset 1
