# Supplementary material for: Molecular Combing of Single DNA Molecules on the 10 Megabase Scale
Source: Sci Rep. 2016 Jan 19;6:19636. doi: 10.1038/srep19636 (PMC4726065; doi:10.1038/srep19636)
Supplement: Supplementary Information [file srep19636-s1.pdf]

SUPPLEMENTARY INFORMATION

Molecular Combing of Single DNA Molecules on the 10 Megabase Scale

Atanas Kaykov[1], Thibaud Taillefumier[2], Aaron Bensimon[3] and Paul Nurse[1,4]

[1]The Rockefeller University, 1230 York Avenue, New York, NY 10065, USA

[2]Lewis-Sigler Institute for Integrative Genomics, Princeton University, Princeton, New Jersey, USA

[3]Genomic Vision, 80-84 rue des Meuniers, 92220 Bagneux, France

[4]The Francis Crick Institute, Lincoln's Inn Fields Laboratories, London WC2A 3LY, United Kingdom

Kaykov_Supplementary_Figure S1

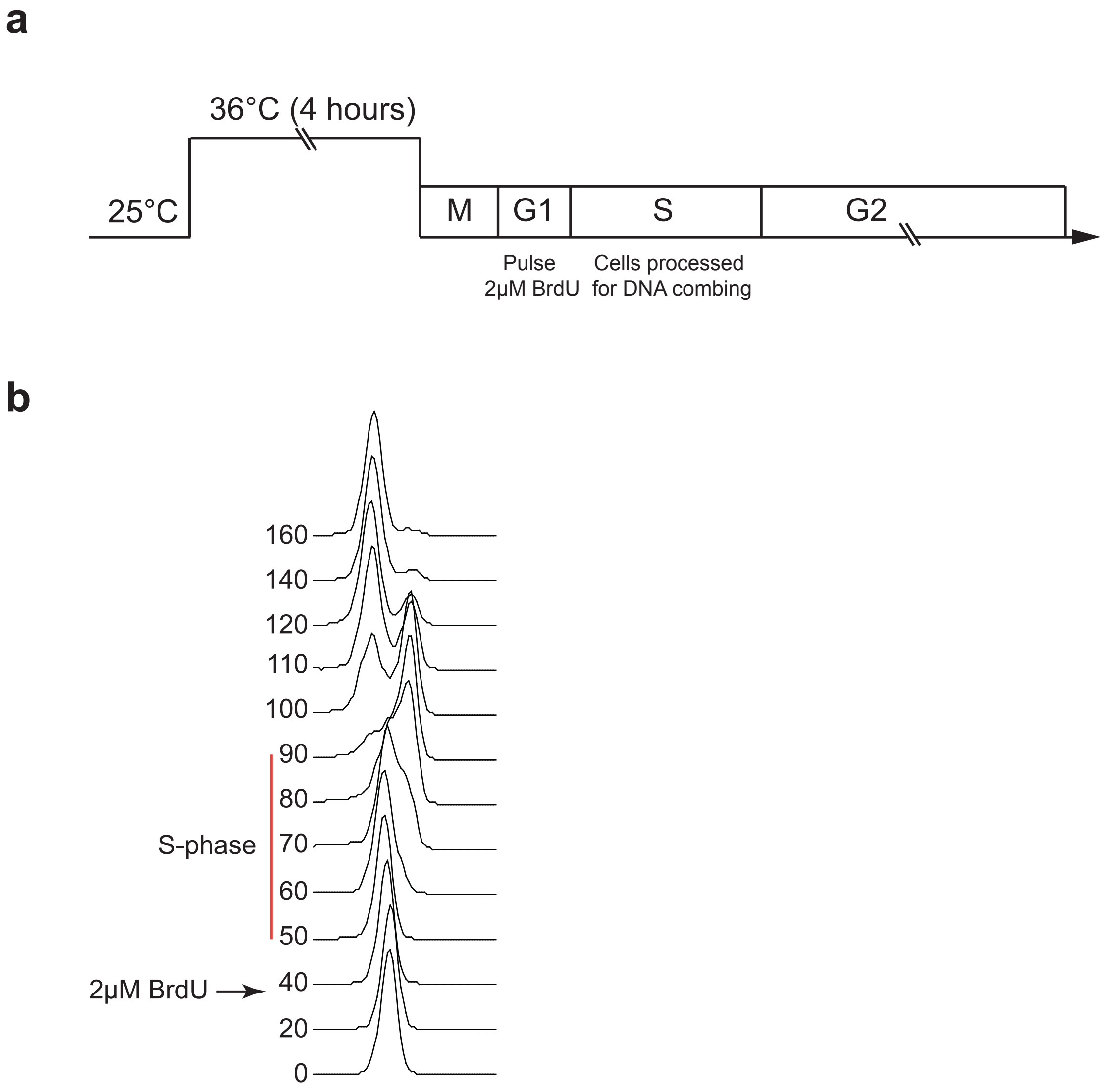

Supplementary Figure S1. **Scheme for *in vivo* DNA labelling of fission yeast cells.**

(a) Fission yeast cells were synchronized at the G2/M transition by blocking *cdc25ts* cells at 36°C followed by temperature shift to 25°C, after which cells proceeded synchronously through mitosis and G1 into S-phase. 2μM BrdU was added to the synchronous cultures at G1 allowing cells to incorporate BrdU into newly synthesized DNA upon entry into S-phase. We have established that DNA synthesis initiates around 45 minutes after release from the temperature block by labeling a *cdc25ts* synchronous cell population with (3H)BrdU at 5 minute intervals and measuring the radioactivity in purified genomic DNA for each time point (data not shown). DNA was prepared for DNA combing from cells 75 minutes after release, corresponding to mid S-phase (estimated from FACS profiles, and (3H)BrdU labeling).

(b) FACS profile of synchronously progressing *cdc25ts* cell population. The numbers on the left side of the FACS profile indicate the time points after release from the temperature block. The time points corresponding to S-phase are indicated with a red line and the time of the BrdU pulse in G1 is indicated with an arrow. Fission yeast cells undergo DNA synthesis before completing cytokinesis, which explains the FACS profile. The peak of septation was estimated to be 70%.

Kaykov_Supplementary_Figure S2

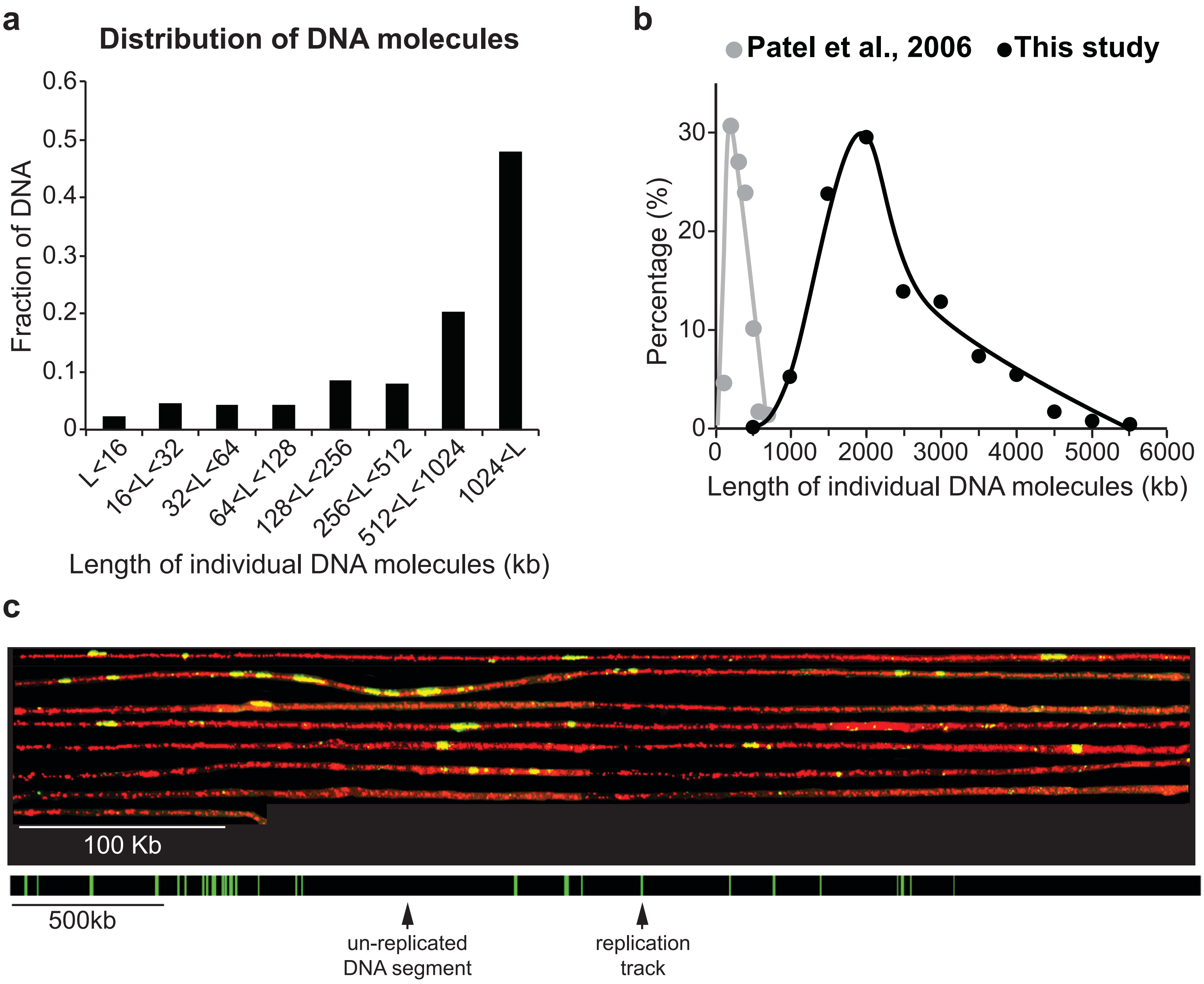

Supplementary Figure S2. Quantifying the enrichment of Mb-scale single DNA molecules prepared from fission yeast.

(a) To quantify the enrichment of single DNA molecules longer than 1Mb, we measured the length of all DNA molecules present in 16 different microscopic fields of view and plotted the fraction of DNA molecules of different lengths.

(b) Comparison between the length of single DNA molecules analysed by Patel et al., 2006 and this study.

(c) A single 4Mb DNA molecule replicated to 5% showing the organisation of replication origin firing in clusters of closely spaced origins. To visualise individual replication tracks the molecule was “cut” in silico into 15 consecutive fragments of 270kb each, and a composite picture was constructed. A representation of the entire 4Mb single DNA molecule is shown below, with green and black bars corresponding to replication tracks and to un-replicated segments of DNA. Black and green bars are drawn to scale.

Kaykov_Supplementary_Figure S3

a

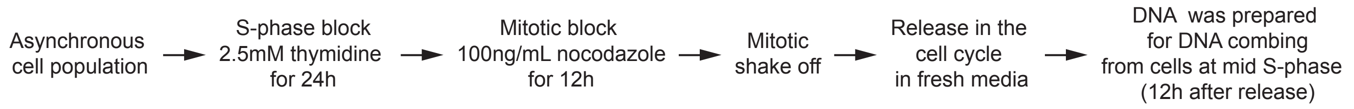

b

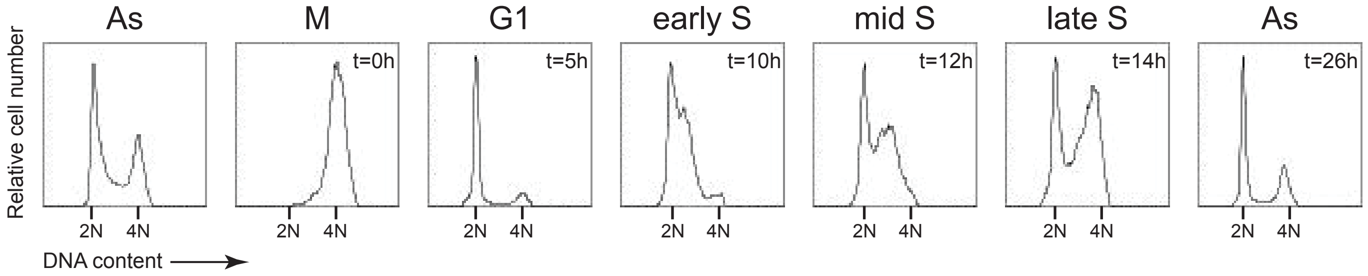

c

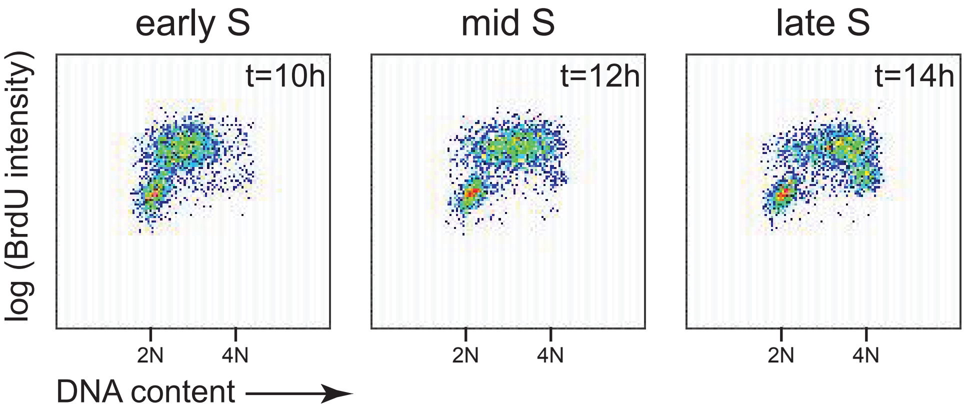

Supplementary Figure S3. **Scheme for *in vivo* DNA labelling of U2OS cells.**

(a) An asynchronous cell population was blocked at the beginning of S-phase using 2.5mM thymidine and then released into 100ng/mL nocodazole to arrest cells in mitosis. Then cells were released from the mitotic block and BrdU was added to the culture after 5 hours (corresponding to G1, see FACS profile below) which allowed cells to incorporate BrdU into newly synthesised DNA upon entry into S-phase. DNA was prepared for DNA combing from cells at 12 hours after release, corresponding to mid S-phase (estimated from FACS profiles).

(b) FACS profile of synchronously progressing U2OS cell population. At release (t=0h) cells were blocked at mitosis and progressed synchronously through mitosis, G1 and S-phase. 10μM BrdU was added 5 hours after release (during G1) allowing cells to incorporate BrdU into newly synthesized DNA upon entry into S-phase. DNA was prepared for DNA combing from cells at 12h after release, during S-phase (estimated from FACS profiles).

(c) To estimate the fraction of cells in early, mid and late S-phase, we pulsed labeled synchronous cell population at the indicated time points with 10μM BrdU for 10 minutes. Cells were ethanol fixed and stained with anti-BrdU antibody and propidium iodite in order to measure the DNA content. We plotted the DNA content on the x-axis and the BrdU intensity on the y-axis. We found that 10h, 12h and 14h after release correspond to early, mid, and late S-phase respectively.

Kaykov_Supplementary_Figure S4

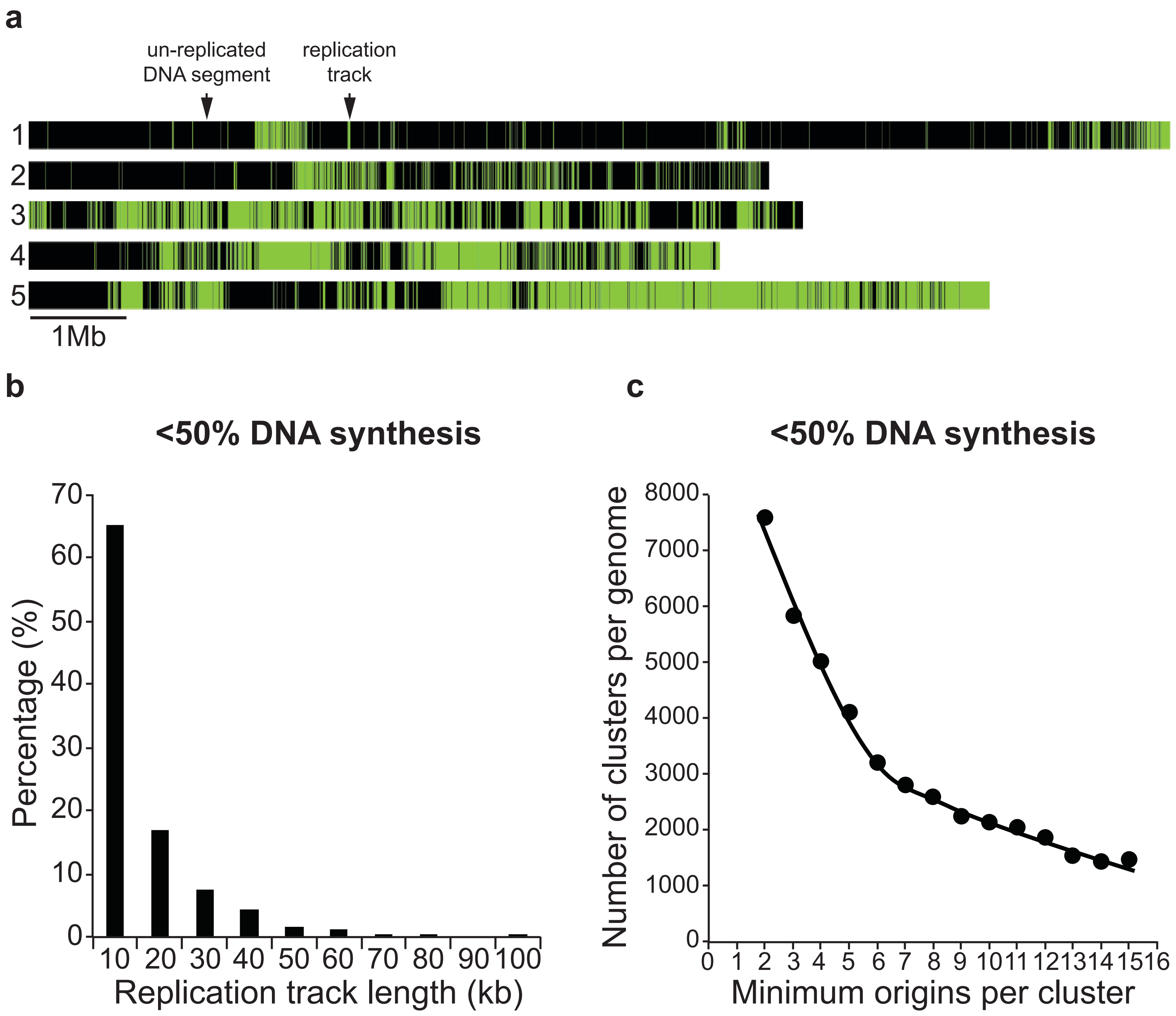

Supplementary Figure S4. Estimate of the minimum number of origins within clusters in U2OS cells.

(a) Five representative single DNA molecules replicated up to 60%. Green bars represent BrdU labelled replication tracks and black bars represent un-replicated segments on DNA molecules. Black and green bars are drawn to scale.

(b) Histogram showing the distribution of replication track length in U2OS cells, measured for molecules replicated up to 50%, (n=664).

(c) Plot of the minimum number of origins fired within 40kb of each other, for molecules replicated up to 50% (45Mb analysed). The data points lay on plot with a descending segment reaching saturation at 6 origins per cluster. This suggests that 6 fired origins is the minimum number of origins defining a cluster in U2OS cells. Assuming that a minimum of 6 fired origins define a cluster, cells having replicated 50% of their DNA will have on average 3200 clusters.

Kaykov_Supplementary_Figure S5

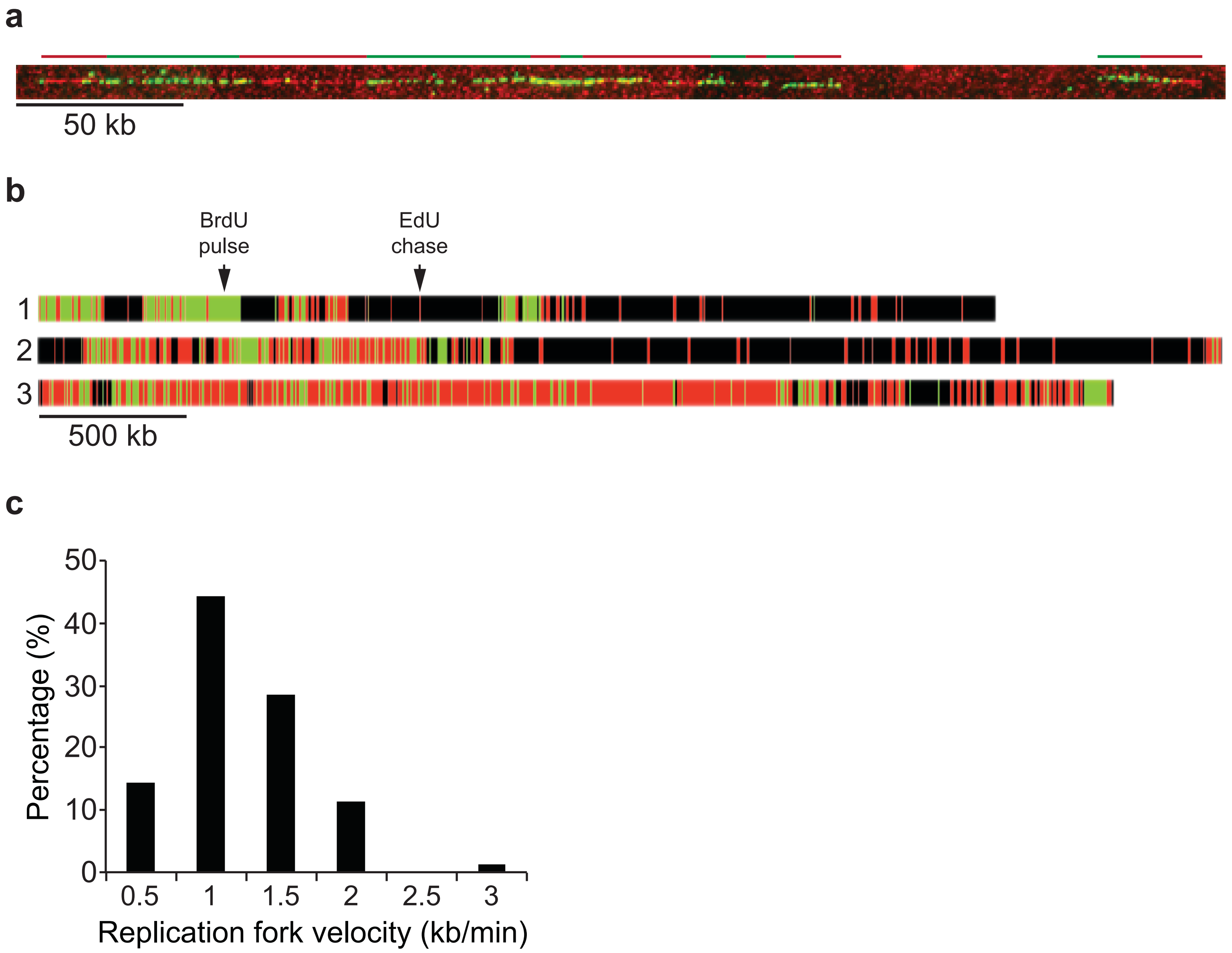

Supplementary Figure S5. Replication fork velocity in human U2OS cells.
Synchronised U2OS cells were pulse labelled with BrdU at the onset of S-phase. At mid S-phase (12 hours after release) BrdU was chased with EdU for 20 minutes and DNA was immediately prepared for DNA combing.

(a) A representative DNA molecule with six replicons. Green (BrdU pulse) and red (EdU chase) lines drawn on top of the molecule depict the position of six replicons. The whole DNA molecule was counterstained with anti-single stranded DNA antibody (not shown).

(b) Three representative single DNA molecules are shown. Black bars correspond to un-replicated regions, green bars correspond to replication tracks labelled with BrdU (pulse) and red bars correspond to replication tracks labelled with EdU (chase). We choose BrdU and EdU thymidine analogues because they can be specifically detected without cross reactivity. Replication fork velocity was derived by measuring the length of EdU labelled replication tracks immediately adjacent to BrdU replication tracks and progressing into un-replicated DNA, divided by the time of incorporation of EdU. The long EdU-tracks on molecule 3 correspond to clusters of closely spaced origins that fire, extend and merge their replication forks during the 20 minute EdU chase.

(c) Histogram showing the distribution of fork velocities in U2OS cells. The average fork velocity is 1kb/min (n=71).

Kaykov_Supplementary_Figure S6

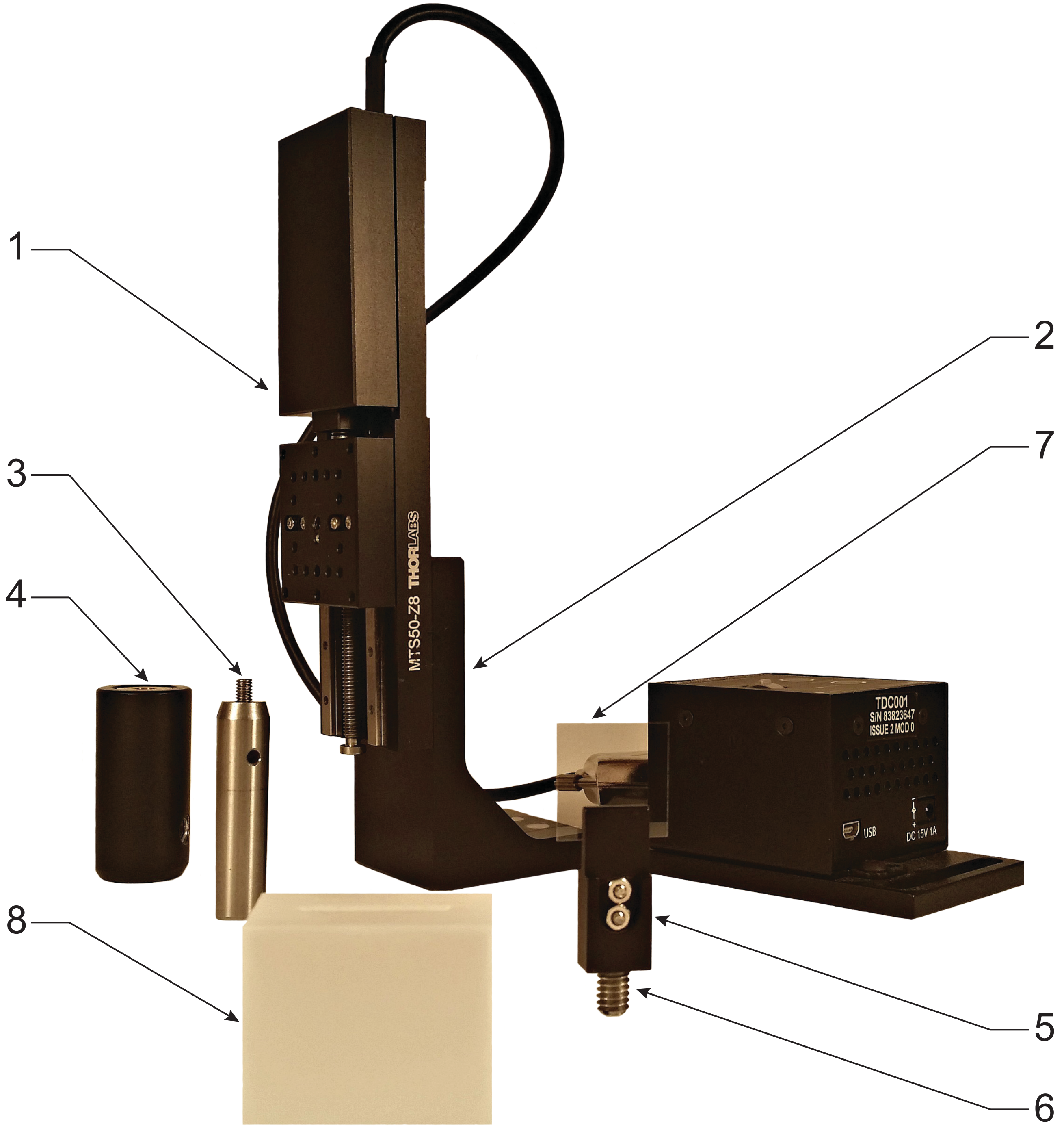

Supplementary Figure S6. Diagram of DNA combing machine.

Individual mechanical modules used to assemble DNA combing machine are shown on the diagram. (**1**) Imperial-Tapped 50mm Motorized Stage with Controller and Power Supply (MTS50-Z8E), (**2**) Right-Angle Bracket for MTS50 Translational Stages (MTS50C-Z8), (**3**) Removable Setscrew (TR2), (**4**) Post Holder Thumbscrew (PH2), (**5**) Compact Dual Holder (DH1), (**6**) Adapter with External Threads (AP8E25E), (**7**) Silane coated glass slide, (**8**) Combing reservoir.

Supplementary Methods

DNA Combing Machine. The DNA combing machine was assembled using precision mechanical modules from ThorLabs including a 50mm motorized linear translational stage (MTS50-Z8) with T-cube DC servo motor controller (TDC001) and a 15 V power supply unit for a single T-cube (TPS001) mounted on a right-angle bracket (MTS50C-Z8) (Supplementary Fig. S6). A removable setscrew (TR2) was screwed onto the translational stage perpendicular to the movement axis and a post holder thumbscrew (PH2) was fitted on the removable screw. The silane coated glass slide was attached to a compact dual holder (DH1) that was screwed parallel to the movement axis on the post holder thumbscrew (PH2) with the aid of an adapter with external threads (AP8E25E). The movement is controlled using apt™ software and PC connected to the T-cube motor controller (TDC001).

Coverslip Silanisation. We used VWR micro cover glass that we placed in ceramic holders (Thomas Scientific), and sonicated in chloroform (Sigma 34854) using ultrasonic water bath cleaner (Branson 200). Glass slides were thoroughly dried using nitrogen gas. Glass slides were activated in plasma cleanser (Harrick Plasma) for 30 seconds and immediately placed in a glass vacuum desiccator (Ted Pella) containing 250μL 7-Octenyltrichlorosilane. We used a rotary vane pump (vaccubrand) to remove air from the chamber creating an atmosphere saturated with 7-Octenyltrichlorosilane vapours, which react with the glass slide. The slides were left for 2 hours within the vacuum desiccator for complete silanisation and stored at RT.

DNA Preparation and Molecular Combing. The cell cycle progression of synchronous fission yeast population was stopped by adding 0.1% NaN_3 then washed in 50mM EDTA pH 7, 0.1% NaN_3 at 4°C. Cells were washed in SP1 buffer (1.2M Sorbitol, 50mM EDTA, 50mM Citrate Phosphate pH 5.6) and recovered in SP1 with 1μg/μL Zymolyase 100T (Seikagaku) and 0.3μg/μL Lysing Enzymes (Sigma) at 37°C. We added equal volume of 2% low melting agarose Mb grade (BioRad) and 0,2% NaN_3 melted in SP1 and equilibrated at 45°C, such that each agarose plug (80μL) contained 1.5-2x10^7 of fission yeast cells. The cell suspension was mixed gently and thoroughly, added to plug molds (BioRad) and incubated at 37°C until 100% “ghost” formation (tested with 10% SDS). Agarose blocks were placed at 4°C for 10 minutes to solidify, ejected in Digestion Buffer

(1% *N*-Laurouylsarcosine, 1mg/ml Proteinase K, 125mM EDTA pH 9.5) and incubated for 1 hour at 50°C. The DB was changed and the plugs were incubated in fresh DB for 48 hours at 50°C, changing the buffer twice more. The plugs were washed for two days in TE 1X pH 7.5 with 100mM NaCl (buffer changes) and melted in MES 50mM pH 6 with 100mM NaCl within combing reservoirs for 15 minutes at 70°C. Combing reservoirs were cooled down to 42°C before adding 2µl β agarase (New England BioLabs) without mixing and incubated overnight at 42°C. Combing reservoirs were cooled down to RT and genomic DNA was combed onto silanised glass surfaces using the combing machine at a speed of 900µm/sec. DNA was dehydrated by incubating slides 2 hours at 65°C and stored at -20°C.

Synchronously progressing U2OS cells were washed three times in PBS at 4°C and recovered in Cell Suspension Buffer (CSB) (10mM Tris-HCl pH=7.5, 20mM NaCl, 50mM EDTA). We added an equal volume of 2% low melting agarose Mb grade (BioRad) with 0,2% NaN_3 melted in CSB and equilibrated at 45°C, such that each agarose plug (80µL) contained 1-2x10^5 U2OS cells. The cell suspension was mixed gently and thoroughly, added to plug molds (BioRad) and placed at 4°C for 10 minutes to solidify. Agarose plugs were ejected into DB (1mg/ml Proteinase K, 1% *N*-Laurouylsarcosine, 0.2% Na Deoxycholate, 100mM EDTA, 10mM Tris-HCl pH 7.5) and incubated for 1 hour at 50°C. The DB was changed and the plugs were incubated at 50°C for 48 hours, changing the buffer twice more. The plugs were washed for two days in TE 1X pH 7.5 with 100mM NaCl before melting them in combing reservoirs and adding 2µl β agarase (New England BioLabs) without mixing and incubated overnight at 42°C.

FISH Probe Hybridisation and Immunodetection. Glass slides with combed DNA were incubated in 1M NaOH for 30minutes in a coplin jar to denature DNA molecules. Slides were washed twice in PBS at 4°C and dehydrated with successive washes in 70%, 90% and 100% ethanol at 4°C. DNA probes were biotin labelled using the BioPrime DNA Labelling System (Invitrogen) and labelled DNA was recovered in Hybridisation Buffer (HB) (50% Formamide, 5XSSC, 0.1% Sarcosine, 0.02% SDS, 2X blocking solution (Roche)). 18µL of labelled DNA probes in HB (~200ng/µL) was added to every slide with denatured DNA molecules, covered with clean coverslip and incubated in a humid chamber for 16-20 hours. Slides were washed three times in 50% Formamide with 2X SSC followed by three washes in 2X SSC for 5 minutes each. Slides were washed in PBS and incubated with 35µM blocking solution (Roche) for 1hour at

37°C in a humid chamber. FISH probes were detected with streptavidin Alexa Fluor 594 (Invitrogen). To detect and orient specific DNA sequences on combed DNA molecules, we used two sets of probes with a unique signature for each chromosomal region represented by differing lengths and distances between them. DNA was detected with mouse anti-single stranded DNA antibody (Millipore), and Alexa Fluor 488 goat anti-mouse antibody (Invitrogen). BrdU epitopes were detected with mouse anti-BrdU antibody (BD Biosciences) and DyLight 405 rat anti-mouse antibody (Jackson ImmunoResearch) and DyLight 405 goat anti-rat antibody (Jackson ImmunoResearch). For the pulse chase experiments, BrdU was detected with Alexa Fluor 488 mouse anti-BrdU antibody (clone MoBU-1, Life Technologies) and Alexa Fluor 488 goat anti-mouse antibody (Invitrogen), and EdU was detected using a Click-iT EdU Alexa Fluor 594 Imaging Kit (Invitrogen). Slides were washed three times with PBS with 0.5% Tween after incubation with each antibody.

Visualisation of Replication Patterns on Multi-Mb Long DNA Molecules. First, we formatted the measured distances on single DNA molecules in Excel files, such that one column contains the distances and the second column contains the color corresponding to the measured DNA segments. Up to 6 colors can be used to generate bar code diagrams: black (N), red (R), green (G), blue (B), cyan (C), yellow (Y), and magenta (M). For instance, a single DNA molecule starting at position 300kb (from the left telomere) having three segments of 30kb, 50kb, and 70kb, and successively colored in red, blue, and black, shall be entered as follows in an Excel file:

	Column A	Column B
Line 1	300	A
Line 2	30	R
Line 3	50	B
Line 4	70	N

In the first line, the letter A indicates the starting position. For other lines, the first column contains the lengths of successive DNA segments and the second column indicates the corresponding color.

Adding another DNA molecule requires adding two new columns, for instance:

	Column A	Column B	Column C	Column D
Line 1	300	A	450	A
Line 2	30	R	20	R
Line 3	50	B	30	Y
Line 4	70	N		

Saving the file in comma separated format (.csv) generates a text file that can be handled by our C routine.

Second, to generate the executable, we downloaded the source code makeBarcode.c in a new working directory and compiled it via a command window as follows. On a Unix machine, make sure you have a C standard compiler, e.g gcc or cc, and enter the following in the command line:

```
gcc  -o makeBarcode.exe makeBarcode.c
```

or

```
cc  -o makeBarcode.exe makeBarcode.c
```

On a Windows machine, enter the following in the command line:

```
cl makeBarcode.c
```

Both of these operations create the executable file makeBarcode.exe.

Finally, running the executable requires a formatted input file, e.g. “data.csv”, saved in the working directory. To run the executable on a Unix machine, enter the following in the command line:

```
./makeBarcode.exe     data.csv     barcode.eps     8    22
```

or

```
./makeBarcode.exe      data.csv       barcode.eps
```

In the first line, the executable takes four arguments: the name of the input file, the name of the output file, the width of a single barcode, and the vertical space between barcodes. The second line only takes the name of the input and output files as arguments, using default values for the barcode width (6) and for the barcode spacing (20). To run the executable on a Windows machine, enter the following in the command line:

```
makeBarcode      data.csv      barcode.eps      8     22
```

or

```
makeBarcode      data.csv       barcode.eps
```

This creates a barcode diagram barcode.eps, written in vectorial Postscript format (.eps), which can be read and edited via standard image viewers such as Preview, Ghostview or Adobe Illustrator.
